# The Biological Effect of Large Single Doses: A Possible Role for Non-Targeted Effects in Cell Inactivation

**DOI:** 10.1371/journal.pone.0084991

**Published:** 2014-01-22

**Authors:** Marlon R. Veldwijk, Bo Zhang, Frederik Wenz, Carsten Herskind

**Affiliations:** Department of Radiation Oncology, Universitätsmedizin Mannheim, Medical Faculty Mannheim, University of Heidelberg, Mannheim, Germany; University Health Network, Canada

## Abstract

**Background and Purpose:**

Novel radiotherapy techniques increasingly use very large dose fractions. It has been argued that the biological effect of large dose fractions may differ from that of conventional fraction sizes. The purpose was to study the biological effect of large single doses.

**Material and Methods:**

Clonogenic cell survival of MCF7 and MDA-MB-231 cells was determined after direct X-ray irradiation, irradiation of feeder cells, or transfer of conditioned medium (CM). Cell-cycle distributions and the apoptotic sub-G1 fraction were measured by flow cytometry. Cytokines in CM were quantified by a cytokine antibody array. γH2AX foci were detected by immunofluorescence microscopy.

**Results:**

The surviving fraction of MCF7 cells irradiated in vitro with 12 Gy showed an 8.5-fold decrease (95% c.i.: 4.4–16.3; P<0.0001) when the density of irradiated cells was increased from 10 to 50×10^3^ cells per flask. Part of this effect was due to a dose-dependent transferrable factor as shown in CM experiments in the dose range 5–15 Gy. While no effect on apoptosis and cell cycle distribution was observed, and no differentially expressed cytokine could be identified, the transferable factor induced prolonged expression of γH2AX DNA repair foci at 1–12 h.

**Conclusions:**

A dose-dependent non-targeted effect on clonogenic cell survival was found in the dose range 5–15 Gy. The dependence of SF on cell numbers at high doses would represent a “cohort effect” in vivo. These results support the hypothesis that non-targeted effects may contribute to the efficacy of very large dose fractions in radiotherapy.

## Introduction

With the rise of novel radiotherapy techniques such as stereotactic radiosurgery (SRS) [1], [2], stereotactic body radiation therapy (SBRT) [3], high-dose-rate (HDR) brachytherapy boost [4], and intra-operative radiotherapy (IORT) [5], [6], irradiation with a single or very few, very large dose fractions is becoming more frequently used. It has been argued that the biological effect of large doses (>∼10 Gy) may be different from that predicted from the response to multiple fractions of 1.8–3 Gy commonly used in radiotherapy. Thus vascular damage and immunological effects may increase the antitumoural efficacy of large doses [7]–[9]. On the other hand, the surviving fraction of cells after high doses may be higher than predicted by the continuously downward bending curve described by the linear-quadratic (L-Q) model for cell inactivation [10]. Indeed, the dose range for which this model can be used is a matter of debate [8], [11].

Radiation-induced bystander effects (BE) have been established as a significant contribution to cell killing and mutation at low doses [12]–[16]. A recent review on intercellular signalling in human exposure scenarios restricts the definition of genuine BEs to effects on unirradiated cells within a volume irradiated with very low doses; effects outside the irradiated volume are termed “abscopal effects”, and effects on irradiated cells caused by other irradiated cells within the target volume are termed “cohort effects” [17]. Although a dose-effect relationship exists at doses below 1–2 Gy, the consensus is that non-targeted radiation effects involving intercellular signalling are saturated in the dose range 1–5 Gy [18]–[22]. Recently, a role of the BE in fractionated radiotherapy with standard or reduced fraction sizes has been proposed [19]. However, doses larger than 10 Gy, relevant for very large fraction sizes, have rarely been studied.

The purpose of the present work was to study the biological effect of large single doses on tumour and normal cells *in vitro*. The clonogenic survival of MCF7 breast cancer cells after a dose of 12 Gy was found to decrease when the number of cells was increased suggesting a role for non-targeted effects of large single doses. Part of this effect was reproduced by a transferrable factor in MCF7 and the breast cancer cell line MDA-MB-231. These findings contribute to understanding the biological effect of very large dose fractions and may have implications for extrapolations using the L-Q model.

## Materials and Methods

### Cell culture

The human breast carcinoma cell lines MCF7, MDA-MB-231, and the mink lung epithelial cell line Mv1Lu (all from American Type Culture Collection, LGC Standards GmbH, Wesel, Germany) were cultured in DMEM supplemented with 10% fetal bovine serum (FBS; all from Biochrom AG, Berlin, Germany). All cells were maintained in a humidified incubator at 37°C under 5% CO_2_ in air. Clonogenic cell survival was determined by the colony formation assay (CFA). Subconfluent cultures of cells were trypsinized and 100–50,000 cells/flask were seeded into triplicate flasks for each dose 5–6 h before irradiation. After irradiation, cells were incubated for colony formation, fixed and stained as described [23]. Clones containing at least 50 cells were scored as a colony, and the surviving fraction (SF) was calculated as ratio of colony yields per seeded cells in cultures irradiated with dose, D, and unirradiated controls: SF = (no. of cols./no. of cells)_D_/(no. of cols./no. of cells)_0Gy_. Feeder cell experiments were performed by seeding 500–50,000 cells per flask in triplicate flasks 5–6 h before irradiation. The flasks were irradiated with a dose of 20 Gy to prevent cell proliferation, and unirradiated cells (usually 100 cells/flask) were seeded and incubated to form colonies. In experiments with conditioned medium (CM), cells were seeded in standard culture medium at 100 cells/flask (0 Gy) or 500 cells/flask (4 Gy) and allowed to attach for 5–6 h before irradiation or sham irradiation. The medium was then removed, replaced by CM produced as described below, and the flasks were incubated for colony formation.

The effect of transforming growth factor (TGF)-β1 (recombinant human, PeproTech GmbH, Hamburg, Germany) on proliferation in mass culture was tested using the vital stain alamarBlue (Invitrogen, Life Technologies,GmbH, Darmstadt, Germany). Reconstitution of activated TGF-β1 was performed according to the supplier's instructions.

### Conditioned medium (CM)

For production of conditioned medium (CM), cells were harvested at approximately 75% confluency and seeded into T75 cell culture flasks at 1×10^6^ cells/T75, unless otherwise noted. After 15 h, the medium was changed and flasks were irradiated or sham irradiated. CM was harvested after 24 h incubation, filtered though a 0.22 µm filter, and used directly for a medium change in the recipient flasks.

### Irradiation

All irradiations were performed with 6 MV X-rays from a linear accelerator (Elekta Synergy, Crawley, UK) at a dose rate of approximately 6.67 Gy/minute. Dosimetry was performed by the physicists of the radiotherapy department as part of the daily quality checks.

### Flow cytometry

The cell cycle distribution and the apoptotic sub-G1 fraction were determined by flow cytometry according to the Nicoletti method as described [24]. In brief, at least 1×10^6^ cells per sample were harvested 24 h after treatment, washed in PBS and fixed overnight in 70% ice-cold ethanol. Cells were washed, resuspended in fresh PBS, and 20 µg/ml RNAse A and 16.7 µg/ml propidium iodide (both Sigma-Aldrich) was added and incubated 30 min at room temperature. Flow cytometry (FACSCalibur, Becton-Dickinson) was performed with doublet-discrimination and FlowJo 7.6.4 software (Tree Star, Ashland, OR, USA) was used for data analysis.

### Cytokine detection

For the identification of potential radiation-induced cytokines released into CM, 1 ml of CM was analysed using the Human Cytokine Array Kit (Panel A, R&D systems, Wiesbaden-Nordenstadt, Germany) according to the manufacturer's instructions. The pixel density was determined using ImageJ software (NIH, Bethesda, MA, USA). The background was subtracted and signals were normalized to the mean of the positive controls on each array.

### Detection of γ-H2AX foci

Exponentially growing cells were seeded into chamber slides (BD Falcon, Heidelberg, Germany) at 5×10^3^ cells per well and treated as described in the results section. At the specified times after irradiation, samples were fixed in 3.7% formaldehyde with 0.2% Triton-X-100, blocked with 1% bovine serum albumin for 30 min and incubated for 1 h with mouse anti-γH2AX antibody (Millipore, Schwalbach, Germany) at room temperature. The cells were washed three times with PBS for 10 min, and then incubated with FITC-conjugated goat-anti-mouse secondary antibody (Millipore) for 1 h, washed and mounted. 50–100 cells were scored for each condition.

### Statistical analysis

At least 3 independent experiments were performed unless otherwise noted. Data are given as mean values ± standard errors (SE). Cell survival curves were fitted by the L-Q model: −ln(SF) = αD+βD^2^, using the non-linear regression tool of SigmaPlot11.0 (Systat Software GmbH, Erkrath, Germany). Least squares regression, ANOVA, and paired t-test were performed with JMP9 statistical software (SAS Institute GmbH, Böblingen, Germany).

## Results

**Figure 1:**
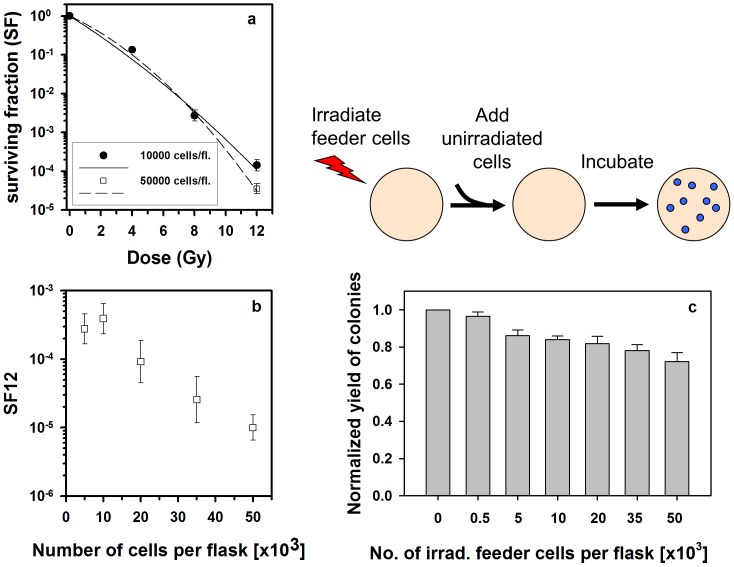
Effect of cell density on cell survival after high-dose irradiation. (a) Influence of the number of seeded cells on the shape of the survival curve of MCF7 cells in the colony formation assay. A significant decrease (P<0.0001) in surviving fraction (SF) after 12 Gy was found when seeding 50,000 cells/T25 flask (open symbol) compared with 10,000 cells/T25 flask. This resulted in an increased downward curvature of the survival curve (n = 7). (b) SF after 12 Gy (SF12) for different numbers of seeded cells (MCF7: 5–50×10^3^/T25 flask) confirmed a significant inverse correlation (P<0.0001) with cell numbers (n = 5). (c) Feeder cell experiment to test the effect of high-dose (20 Gy) irradiated cell numbers on colony formation of unirradiated cells (MCF7). The experimental design is shown above the data diagram. A significant inverse correlation (P<0.0001) between the number of irradiated feeder cells per T25 flask and the normalized plating efficiency of unirradiated MCF7 cells was observed (n = 3). Mean±standard errors are shown.

The clonogenic survival after direct irradiation of MCF7 cells was determined in the dose range 4–12 Gy. In order to improve the accuracy of SF values at 12 Gy (SF12), two different cell numbers per flask were seeded. Unexpectedly, increasing the cell number from 10 to 50×10^3^ cells/flask resulted in a strong decrease in SF (Figure 1a). This affected the shape of the fitted survival curve, yielding a stronger downward curvature when more cells were seeded. In order to verify the observed inverse relation between SF12 and cell numbers, different numbers were irradiated with 12 Gy. These experiments showed a significant decrease of SF12 (P = 0.0001) with increasing numbers of cells per flask in the range 5–50×10^3^ (Figure 1b) corroborating the dependence of SF12 on the cell density shown in Figure 1a. Although the number of colonies after irradiation with 12 Gy was small (mean of all 12 experiments was ∼2.8 colonies per triplicate flask at 50,000 cells/flask) the logarithmic transformation of SF12 was approximately normal distributed. Analysing ln(SF12) at 10 and 50×10^3^ cells/flask for all 12 experiments together, the (geometrical) mean decrease of SF12 was 8.5-fold (95% c.i.: 4.4–16.3; P<0.0001) corresponding to a reduction by 88% for a 5-fold increase in the density of irradiated cells. The plating efficiency (PE) of unirradiated MCF7 cells (mean PE = 53%) did not influence this analysis since the same PE value was used in each experiment to calculate SF12 for the different cell numbers per flask.

In order to test whether irradiated cells inhibit colony formation of unirradiated cells, a feeder cell experiment was performed. One hundred unirradiated cells per flask were plated into flasks containing different numbers of cells irradiated with 20 Gy prior to seeding the test cells. This dose was chosen to rule out formation of colonies by surviving irradiated cells. A significant inverse correlation (P<0.0001) was found between the normalized yield of colonies of unirradiated cells and the number of irradiated feeder cells in the range 5–50×10^3^ per flask (Figure 1c) with a maximum reduction of 28% (95% c.i.: 22–34).

**Figure 2:**
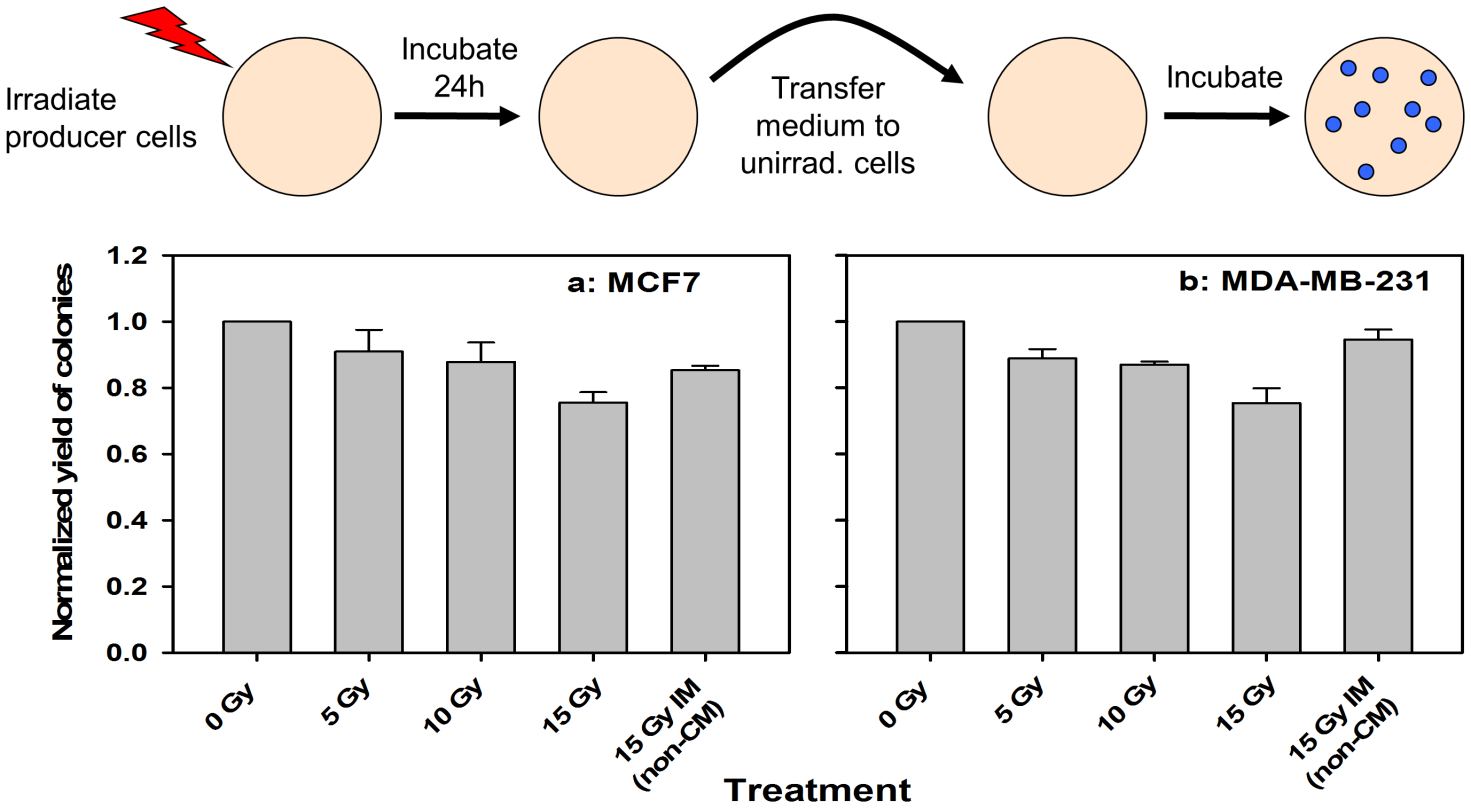
Medium transfer experiments to test effect of transferrable factor. The experimental design is show above the diagrams. The effect of incubation with conditioned medium (CM) from irradiated cells on the clonogenic growth of unirradiated recipient cells for MCF7 (a) and MDA-MB-231(b). A dose-dependent decrease was found for MCF7 (P = 0.0001) and MDA-MB-231 (P<0.0001) compared to the CM from unirradiated cells. 15 Gy IM (non-CM): medium irradiated without cells. Mean±standard errors (n = 4) are shown.

To test whether a transferable factor was involved, the effect of conditioned medium (CM) from MCF7 cells irradiated with different doses (D = 5–15 Gy) was tested on unirradiated recipient MCF7 clonal cultures. A significant dose dependence (P = 0.0001) of the normalized yield of colonies was observed with a maximum decrease of 24% (95% c.i.: 15–34) at 15 Gy (Figure 2a). A highly significant dose-dependent inhibition of colony formation (P<0.0001) was also found for MDA-MB-231 breast cancer cells with a similar maximum decrease of 24% (95% c.i.: 10–38) (Figure 2b). The decrease at 15 Gy relative to 5 and 10 Gy analysed together was significant (or borderline significant) for both breast cancer cell lines (P = 0.053 and 0.007, respectively). For MDA-MB-231, no significant effect of adding irradiated medium (IM: irradiated without cells) to the recipient cells was detected. These results strongly suggested that part of the cell number-dependent decrease in SF after high-dose irradiation is caused by a radiation-induced transferable factor in the CM.

**Figure 3:**
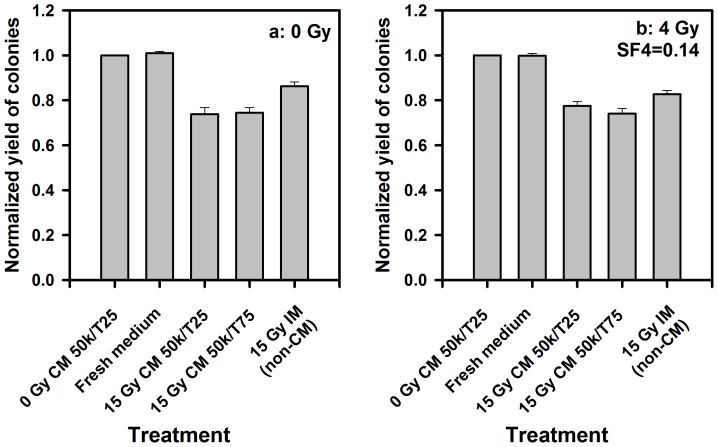
No influence of irradiation of recipient cell on the effect of conditioned medium (CM). Inhibition of colony formation by 15 Gy irradiation CM was similar on unirradiated (a) and 4 Gy irradiated (b) recipient MCF7 cells. The different CM production conditions are shown on the abcissa. The different media showed essentially the same effect on unirradiated and irradiated recipients and were in line with previous experiments (with the exception of the 15 Gy IM (irradiated medium); see text for explanation). In addition, the volume of CM during production does not seem to alter the magnitude of the BE. Mean±standard errors (n = 3) are shown.

The smaller reduction in colony formation by CM compared to the effect seen for irradiation of increasing cell numbers might be explained if the CM influenced repair processes in irradiated recipient cells. To test this hypothesis, CM from 15 Gy irradiated cells was added to recipient cells irradiated with 4 Gy or sham irradiated immediately prior to the medium change (Figure 3a,b). Furthermore, the number of cells in CM producer flasks was reduced to 50×10^3^ per flask, and T25 flask (5 ml of medium) were tested in addition to T75 (15 ml of medium), in order to test if the concentration of the transferable factor was limiting. The results (Figure 3a) reproduced the effect of CM on unirradiated recipient cells from Figure 2a and thus 50×10^3^ cells per flask seemed sufficient. The results for irradiated recipient cells was essentially identical (Figure 3b) and thus the additional cell kill with increasing cell numbers seen in Figures 1a,b did not appear to involve inhibition of repair processes. Furthermore, a reduction in clonogenic growth of cells that did not receive a medium change was observed in the MCF7 cells, indicating that this cell line may be more sensitive to depletion of essential medium components. Thus radiation-induced depletion might be involved in the effect of irradiated medium.

**Figure 4:**
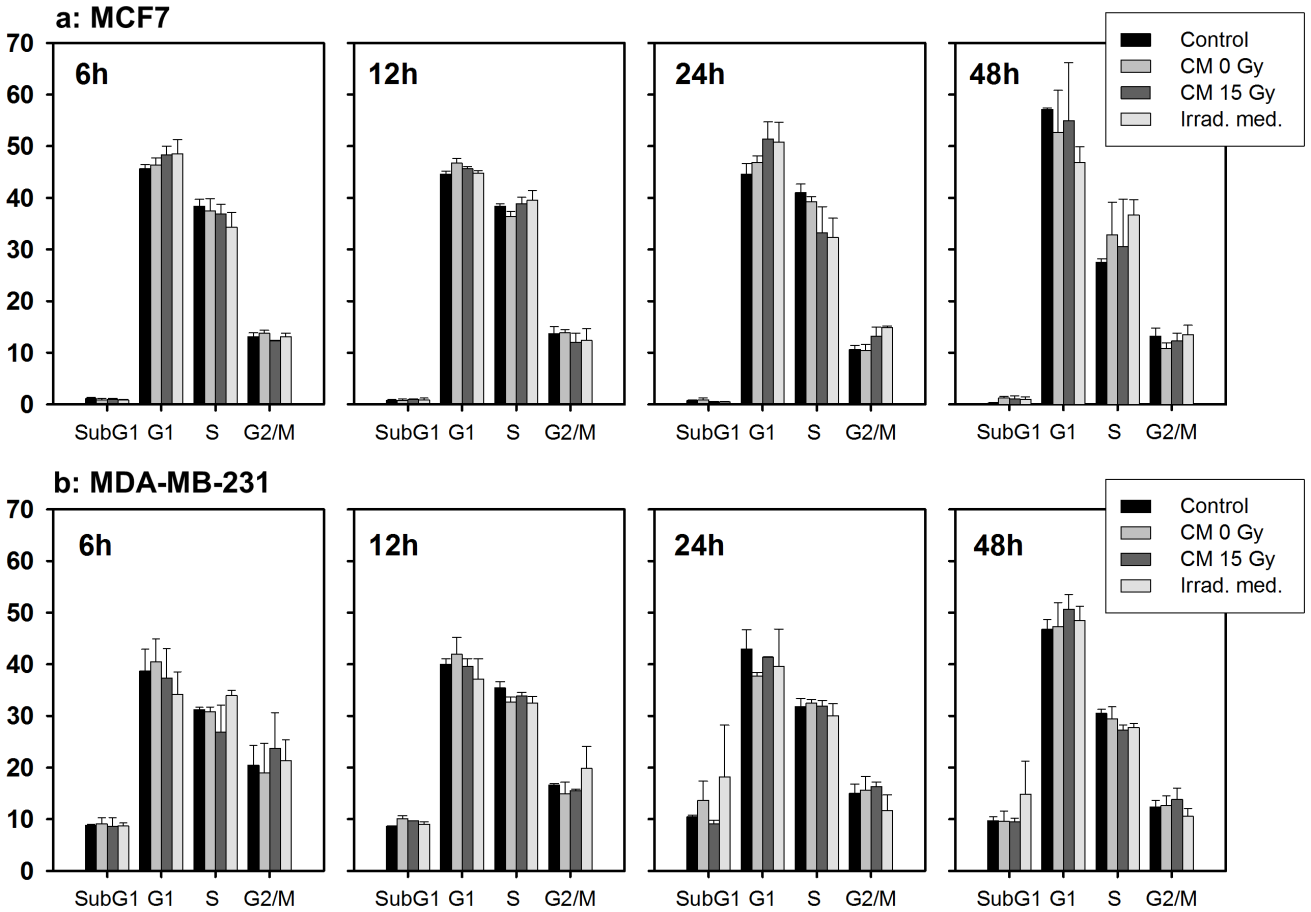
No effect of conditioned medium (CM) on cell cycle distribution and apoptosis. Flow cytometry was performed at different time points after incubation of recipient MCF7 (a) or MDA-MB-231 (b) cells with 15 Gy CM. No effect on the cell cycle distribution or apoptosis rate (sub-G1) was observed at any time point 6–48 h. Mean±standard errors (n = 3) are shown.

Since CM seemed to inhibit clonogenic proliferation, its potential effect on cell cycle distribution and apoptosis was investigated. Unirradiated recipient MCF7 and MDA-MB-231 cells were treated with 15 Gy CM, fixed at different time points (6–48 h) and analysed by flow cytometry (Figure 4a,b). No significant differences in cell cycle distribution and apoptosis rates were observed for the different treatment groups. Thus the effect of CM on clonogenic cell survival did not seem to be caused by apoptosis or changes in cell cycle progression.

**Figure 5:**
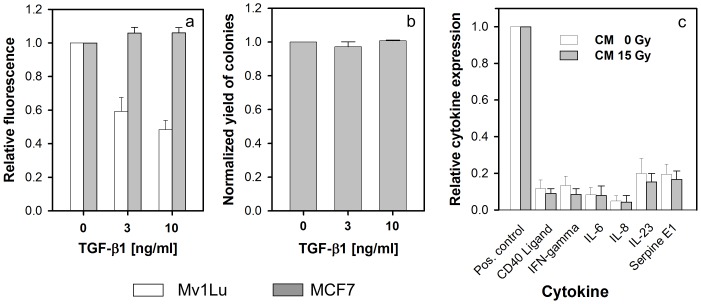
Role of cytokines in intercellular signalling. (a) 3–10 ng/ml TGF-β1 had no effect on proliferation of MCF7 in mass culture (3 days incubation of proliferation test with vital dye alamarBlue). Inhibition of Mv1Lu cells confirmed the activity of TGF-β1. (b) No effect of 3–10 ng/ml TGF-β1 on colony formation of MCF7 cells. (c) Detection of cytokines expressed in CM from 15 Gy irradiated and unirradiated MCF7 cultures. No significant difference was observed (P = 0.46–0.95). The presence of the CM-mediated inhibitory effect on colony formation was verified in each experiment (not shown). Mean±standard errors (n = 3) are shown. None of the complete set of 36 cytokines tested showed a significant difference (P = 0.18–0.96). See Figure S1.

Recently, TGF-β1 was reported to be a key cytokine in down-stream signalling of a low-dose effect of medium transfer in glioma cells [25], [26]. Therefore, we tested the effect of TGF-β1 on proliferation of MCF7 cells. In addition to MCF7 cells, the TGF-β1-sensitive cell line Mv1LU was used to confirm the activity of TGF-β1. In a mass culture proliferation assay, 3–10 ng/ml of TGF-β1 did not inhibit proliferation of MCF7 cells, in fact a weak but significant stimulation (P = 0.005) was observed. Inhibition of Mv1Lu cells confirmed the activity of TGF-β1 (Figure 5a). Colony formation of MCF7 cells was not inhibited by TGF-β1 either (Figure 5b).

In order to test whether other cytokines might be involved (e.g. IL-6, IL-8 [27], [28]), a cytokine antibody array detecting 36 different cytokines was used for profiling the cytokine expression in CM from cultures irradiated with 15 Gy compared with unirradiated producer cells. Several cytokines were found in CM from both cultures but no significant difference between CM from irradiated and unirradiated cultures was observed (P = 0.18–0.96). The expression of CD40 ligand, IFN-γ, IL-6, IL-8 IL-23, and Serpin E1 are shown in Figure 5c. The results of the complete arrays are shown in Figure S1. The inhibitory effect of 15 Gy irradiated CM on colony formation was verified in each experiment.

**Figure 6:**
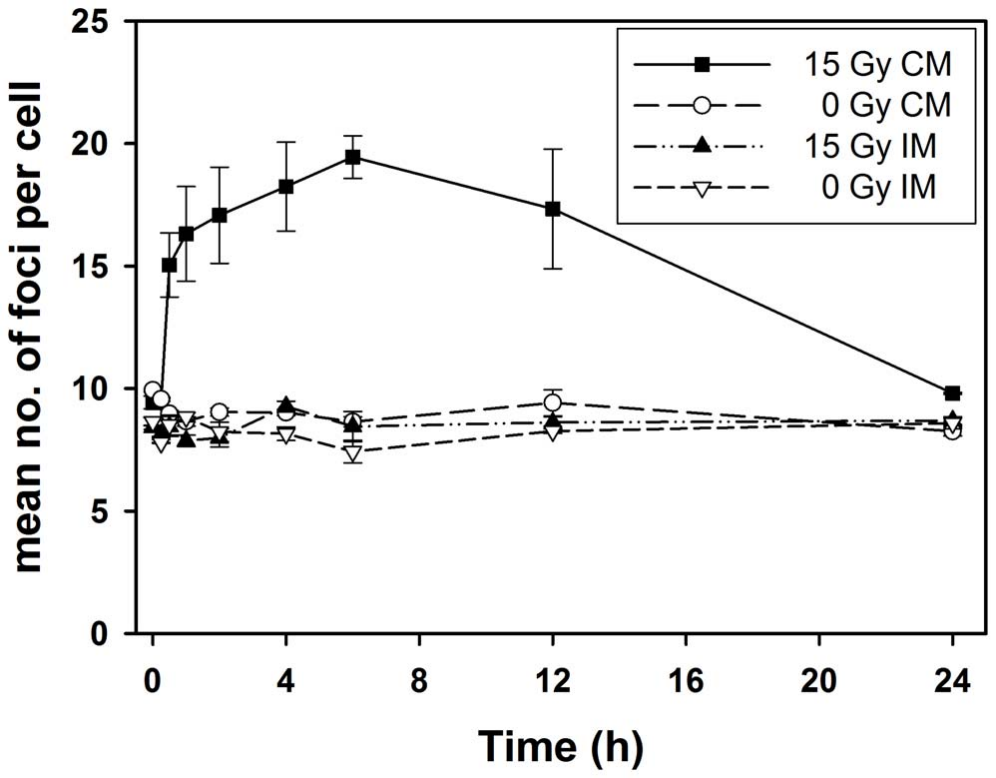
Conditioned medium (CM) induces prolonged γ-H2AX foci in MCF7 cells. Recipient cells were incubated with conditioned medium (CM) from 15 Gy or sham irradiated MCF7 cells, or with 15 Gy irradiated or sham irradiated medium (IM). Treatment with 15 Gy irradiated CM produced rapid induction of γH2AX foci which stayed elevated up to 6–12 h. The mean number of foci per cell receiving CM from 15 Gy irradiated cells was significantly higher than in cells receiving unirradiated CM, 15 Gy irradiated or unirradiated non-conditioned medium (IM; P<0.0001 for time = 1–12 h) Mean±standard errors (n = 3) are shown.

DNA damage and repair is an important cellular response to irradiation and has previously been suggested to be involved in a low-dose radiation-induced BE [29]. Therefore, γH2AX immunostaining was performed on bystander recipient MCF7 cells which received CM from 0 Gy or 15 Gy irradiated cells or irradiated medium. The addition of 15 Gy CM to the bystander recipient MCF7 cells resulted in a significant (P<0.0001) 2-fold increase in the mean number of foci per cell at 1–12 h, peaking at approximately 6 h (Figure 6). An enhanced level was still present at 12 h whereas the number of foci decreased to nearly background levels at 24 h. By contrast, neither CM from unirradiated cells nor non-conditioned IM caused an increase in the number of foci.

## Discussion

In the present study we have shown that the SF of breast cancer cells after a high single dose (12 Gy) depends on the number of cells irradiated per flask. Part of the effect was shown to be due to a transferable factor capable of reducing clonogenic proliferation of unirradiated breast cancer and endothelial cells. While we did not find evidence for the involvement of TGF-β1 or modulation of 36 cytokines in CM from irradiated cells tested with the cytokine array, the transferable factor clearly induced prolonged expression of γH2AX foci in unirradiated cells, indicating activation of the DNA double-strand break repair machinery.

To our knowledge, the present study is the first to show a significant enhancement of the non-targeted effect of 15 Gy irradiated CM relative to 5–10 Gy. This demonstrates a role of intercellular signalling on cell survival in the high-dose region beyond 10 Gy and supports the notion that high single doses, as used in SRS, SBRT, or IORT, may elicit different biological effects than fraction sizes of 1.8–3 Gy as used in standard or moderately hypofractionated radiotherapy [8], [9], [30].

Although the exact value of the 8.5-fold reduction in SF12 by increasing the cell density from 5 to 50×10^3^ cells/flask had a wide confidence interval owing to the low number of colonies in the irradiated flask, the reduction was reproduced in 12 experiments and was highly significant (P<0.0001). An effect of cell density on SF has previously been described for human fibroblasts and a melanoma cell lines by Pomp et al. [31]. In their study, non-linearity of PE was found to be associated with high numbers of colonies formed and was most likely due to a biased detection of crowded colonies since it was not observed for other cell lines producing small and compact colonies. Furthermore, only a minor influence on SF was observed for fibroblasts, which was due to high number of colonies in the unirradiated control cultures. By contrast, the number of colonies in the present study was low in irradiated flasks, and the size and morphology of MCF7 colonies allowed unbiased detection in unirradiated as well as irradiated flasks. Even if the PE of unirradiated cultures were non-linear it would have no effect on the comparison of SF12 for different cell densities because the same PE was used to calculate SF12 in each experiment.

The inhibitory effect on colony formation in the feeder cell and medium transfer (CM) experiments was approximately one third of the inhibition (up to 88%) seen when all cells were irradiated with 12 Gy. At the cell density used here, cell-cell contacts seem unlikely. Furthermore, the feeder cell experiment ruled out long-term effects via cell-cell contacts being responsible for the difference although it cannot be ruled out that a short-lived signal from the irradiated cells might decay before seeding the unirradiated cells. Fast kinetics experiments were not possible in our experimental setup, so we chose to focus on the effect of medium transfer.

Historically, the term BE has been used to describe a wide variety of non-targeted effects [32]. Inter-cellular signaling via CM in medium transfer experiments has been studied mainly in the low-dose region below 1–5 Gy. In most cases, a dependence on dose has been observed only below 1–2 Gy with saturation at 1–5 Gy [18]–[22]. A recent study suggested a possible contribution from a bystander effect in radiotherapy with fraction sizes up to approximately 5 Gy [19]. Previously a bystander effect mediated by TNF-α has been described in lung cancer cells treated with CM from 10 Gy relative to 2 Gy irradiated cultures [33]. On the other hand, Gow et al. found a reversal of the BE at 10 Gy in immortalized human keratinocytes, which was interpreted to be related to a saturation of the signal-to-recipient cell number [25], [34]. However, in our system, we saw an enhancement of the non-targeted effect at high doses even though the ratio of producer cell-to-recipient cell number was two orders of magnitude higher than in the keratinocyte system [34].

Recent work implicated TGF-β1 as a downstream mediator of low-dose BEs for inactivation of glioma cells treated with CM from cultures irradiated with high-energy electrons [25], [35]. An earlier study also found a TGF-β1-mediated low-dose BE of CM from lung fibroblasts irradiated with α-rays although, in these cells, TGF-β1 stimulated proliferation [26]. However, in the present study TGF-β1 did not inhibit proliferation or clonogenicity of MCF7 cells. Thus our results did not support a role of TGF-β1 in inhibiting colony formation by CM from cells irradiated with 5–15 Gy. Medium transfer experiments of CM from cells irradiated with low doses of gamma- or X-rays have implicated cytokines such as IL-6 or IL-8 [27], [28]. However, we did not find any significant differences in the expression of 36 cytokines in the CM from high-dose irradiated and unirradiated cells. Notably, TNF-α, which has been implicated in inducing apoptosis in some types of bystander cells [33], [36], was not expressed at measurable levels in CM regardless of dose.

The prolonged induction of γH2AX foci by CM from high-dose irradiated cells is consistent with previous findings at lower doses and may indicate complex DNA repair processes [37]. Thus γH2AX foci have been found to represent secondary DSB formed in an ATR-dependent manner at stalled replication forks where single-stranded DNA is exposed [37], [38]. While radiation-induced oxidative damage was considered responsible for the stalled replication forks in these studies, short-lived radiation-induced radicals are unlikely to be involved in γH2AX foci induction by CM in the present work because CM was collected 24 h post-irradiation. Furthermore, medium irradiated without cells did not induce γH2AX foci in MCF7. The moderate inhibitory effect of irradiated medium on colony formation observed for MCF7 (Figure 2a) but not MDA-MB-231 (Figure 2b), might be due to a higher sensitivity to radiation-induced depletion of factors in the medium required for proliferation.

The increased downward curvature of the cell survival curve by increasing the cell density (Figure 1a,b) shows that the shape of *in vitro* survival curve can be influenced by experimental factors that might also be relevant *in vivo*. If a similar effect exists *in vivo*, this would represent a “cohort effect” as defined by Blyth and Sykes [17]. Thus, conceivably cohort effects, such as the cell density effect, might neutralize the deviation of cell survival curves from linear-quadratic shape at high doses, which has been used as an argument against applying this model outside the dose range 1–8 Gy [8], [10], [11]. In summary, the present work supports the hypothesis that the biological effect of very large dose fractions differ from the effect of conventional fraction sizes. We suggest that non-targeted effects, including the cohort effect described here, may enhance the efficacy of very large dose fractions in radiotherapy.

## Supporting Information

Figure S1 **Relative protein levels in CM of all 36 cytokines on cytokine array.** Mean values±standard errors of normalized signals of cytokines in CM from 15 Gy irradiated and unirradiated MCF7 cultures from three independent experiments are shown. No significant differences were observed between CM from irradiated and unirradiated cultures (P = 0.18–0.96; n = 3). MIF and RANTES showed high expression levels with considerable variation in between experiments (outliers). The variation did not appear to be limited to CM from irradiated cells (see data for RANTES).
(TIF)

## References

[1] Hof H, Muenter M, Oetzel D, Hoess A, Debus J, et al (2007) Stereotactic single-dose radiotherapy (radiosurgery) of early stage nonsmall-cell lung cancer (NSCLC). Cancer 110: 148–155. 17516437 10.1002/cncr.22763

[2] Kondziolka D, Flickinger JC, Lunsford LD (2012) Radiosurgery for brain metastases. Prog Neurol Surg 25: 115–122. 22236673 10.1159/000331184

[3] Timmerman RD, Kavanagh BD, Cho LC, Papiez L, Xing L (2007) Stereotactic body radiation therapy in multiple organ sites. J Clin Oncol 25: 947–952. 17350943 10.1200/JCO.2006.09.7469

[4] Morton G, Loblaw A, Cheung P, Szumacher E, Chahal M, et al (2011) Is single fraction 15 Gy the preferred high dose-rate brachytherapy boost dose for prostate cancer? Radiother Oncol 100: 463–467. 21924511 10.1016/j.radonc.2011.08.022

[5] Vaidya JS, Joseph DJ, Tobias JS, Bulsara M, Wenz F, et al (2010) Targeted intraoperative radiotherapy versus whole breast radiotherapy for breast cancer (TARGIT-A trial): an international, prospective, randomised, non-inferiority phase 3 trial. Lancet 376: 91–102. 20570343 10.1016/S0140-6736(10)60837-9

[6] Veronesi U, Orecchia R, Luini A, Galimberti V, Zurrida S, et al (2010) Intraoperative radiotherapy during breast conserving surgery: a study on 1,822 cases treated with electrons. Breast Cancer Res Treat 124: 141–151. 20711810 10.1007/s10549-010-1115-5

[7] Flickinger JC, Kondziolka D, Lunsford LD (2003) Radiobiological analysis of tissue responses following radiosurgery. Technol Cancer Res Treat 2: 87–92. 12680788 10.1177/153303460300200203

[8] Kirkpatrick JP, Meyer JJ, Marks LB (2008) The linear-quadratic model is inappropriate to model high dose per fraction effects in radiosurgery. Semin Radiat Oncol 18: 240–243. 18725110 10.1016/j.semradonc.2008.04.005

[9] Herskind C, Wenz F (2009) Is there more to intraoperative radiotherapy (IORT) than physical dose ? Int J Radiat Oncol Biol 74: 976–977.

[10] Garcia LM, Wilkins DE, Raaphorst GP (2007) Alpha/beta ratio: A dose range dependence study. Int J Radiat Oncol Biol Phys 67: 587–593. 17236975 10.1016/j.ijrobp.2006.10.017

[11] Brenner DJ (2008) The linear-quadratic model is an appropriate methodology for determining isoeffective doses at large doses per fraction. Semin Radiat Oncol 18: 234–239. 18725109 10.1016/j.semradonc.2008.04.004 PMC2750078

[12] Buonanno M, de Toledo SM, Azzam EI (2011) Increased frequency of spontaneous neoplastic transformation in progeny of bystander cells from cultures exposed to densely ionizing radiation. PLoS One 6: e21540. 21738697 10.1371/journal.pone.0021540 PMC3125249

[13] Hei TK, Zhou H, Ivanov VN, Hong M, Lieberman HB, et al (2008) Mechanism of radiation-induced bystander effects: a unifying model. J Pharm Pharmacol 60: 943–950. 18644187 10.1211/jpp.60.8.0001 PMC4410683

[14] McMahon SJ, Butterworth KT, Trainor C, McGarry CK, O'Sullivan JM, et al (2013) A kinetic-based model of radiation-induced intercellular signalling. PLoS One 8: e54526. 23349919 10.1371/journal.pone.0054526 PMC3551852

[15] Mothersill C, Seymour C (2012) Are epigenetic mechanisms involved in radiation-induced bystander effects? Front Genet 3: 74. 22629281 10.3389/fgene.2012.00074 PMC3354559

[16] Trainor C, Butterworth KT, McGarry CK, McMahon SJ, O'Sullivan JM, et al (2012) DNA damage responses following exposure to modulated radiation fields. PLoS One 7: e43326. 22912853 10.1371/journal.pone.0043326 PMC3422245

[17] Blyth BJ, Sykes PJ (2011) Radiation-induced bystander effects: what are they, and how relevant are they to human radiation exposures? Radiat Res 176: 139–157. 21631286 10.1667/rr2548.1

[18] Gerashchenko BI, Howell RW (2003) Flow cytometry as a strategy to study radiation-induced bystander effects in co-culture systems. Cytometry A 54: 1–7. 12820115 10.1002/cyto.a.10049

[19] Gomez-Millan J, Katz IS, Farias Vde A, Linares-Fernandez JL, Lopez-Penalver J, et al (2012) The importance of bystander effects in radiation therapy in melanoma skin-cancer cells and umbilical-cord stromal stem cells. Radiother Oncol 102: 450–458. 22169765 10.1016/j.radonc.2011.11.002

[20] Liu Z, Mothersill CE, McNeill FE, Lyng FM, Byun SH, et al (2006) A dose threshold for a medium transfer bystander effect for a human skin cell line. Radiat Res 166: 19–23. 16808607 10.1667/RR3580.1

[21] Tomita M, Maeda M, Maezawa H, Usami N, Kobayashi K (2010) Bystander cell killing in normal human fibroblasts is induced by synchrotron X-ray microbeams. Radiat Res 173: 380–385. 20199223 10.1667/RR1995.1

[22] Yang H, Asaad N, Held KD (2005) Medium-mediated intercellular communication is involved in bystander responses of X-ray-irradiated normal human fibroblasts. Oncogene 24: 2096–2103. 15688009 10.1038/sj.onc.1208439

[23] Liu Q, Schneider F, Ma L, Wenz F, Herskind C (2013) Relative Biologic Effectiveness (RBE) of 50 kV X-rays Measured in a Phantom for Intraoperative Tumor-Bed Irradiation. Int J Radiat Oncol Biol Phys 85: 1127–1133. 22981707 10.1016/j.ijrobp.2012.08.005

[24] Maier P, Fleckenstein K, Li L, Laufs S, Zeller WJ, et al (2006) Overexpression of MDR1 using a retroviral vector differentially regulates genes involved in detoxification and apoptosis and confers radioprotection. Radiat Res 166: 463–473. 16953664 10.1667/RR0550.1

[25] Gow MD, Seymour CB, Ryan LA, Mothersill CE (2010) Induction of bystander response in human glioma cells using high-energy electrons: a role for TGF-beta1. Radiat Res 173: 769–778. 20518656 10.1667/RR1895.1

[26] Iyer R, Lehnert BE, Svensson R (2000) Factors underlying the cell growth-related bystander responses to alpha particles. Cancer Res 60: 1290–1298. 10728689

[27] Facoetti A, Mariotti L, Ballarini F, Bertolotti A, Nano R, et al (2009) Experimental and theoretical analysis of cytokine release for the study of radiation-induced bystander effect. Int J Radiat Biol 85: 690–699. 19637080 10.1080/09553000903020016

[28] Mariotti LG, Bertolotti A, Ranza E, Babini G, Ottolenghi A (2012) Investigation of the mechanisms underpinning IL-6 cytokine release in bystander responses: The roles of radiation dose, radiation quality and specific ROS/RNS scavengers. Int J Radiat Biol 88: 751–762. 22709338 10.3109/09553002.2012.703365

[29] Nagasawa H, Huo L, Little JB (2003) Increased bystander mutagenic effect in DNA double-strand break repair-deficient mammalian cells. Int J Radiat Biol 79: 35–41. 12556329

[30] Truman JP, Garcia-Barros M, Kaag M, Hambardzumyan D, Stancevic B, et al (2010) Endothelial membrane remodeling is obligate for anti-angiogenic radiosensitization during tumor radiosurgery. PLoS One 5: e12310. 20808818 10.1371/journal.pone.0012310 PMC2924400

[31] Pomp J, Wike JL, Ouwerkerk IJ, Hoogstraten C, Davelaar J, et al (1996) Cell density dependent plating efficiency affects outcome and interpretation of colony forming assays. Radiother Oncol 40: 121–125. 8884965 10.1016/0167-8140(96)01767-7

[32] Mothersill C, Seymour C (2001) Radiation-induced bystander effects: past history and future directions. Radiat Res 155: 759–767. 11352757 10.1667/0033-7587(2001)155[0759:ribeph]2.0.co;2

[33] Shareef MM, Cui N, Burikhanov R, Gupta S, Satishkumar S, et al (2007) Role of tumor necrosis factor-alpha and TRAIL in high-dose radiation-induced bystander signaling in lung adenocarcinoma. Cancer Res 67: 11811–11820. 18089811 10.1158/0008-5472.CAN-07-0722

[34] Gow MD, Seymour CB, Byun SH, Mothersill CE (2008) Effect of dose rate on the radiation-induced bystander response. Phys Med Biol 53: 119–132. 18182691 10.1088/0031-9155/53/1/008

[35] Shao C, Folkard M, Prise KM (2008) Role of TGF-beta1 and nitric oxide in the bystander response of irradiated glioma cells. Oncogene 27: 434–440. 17621264 10.1038/sj.onc.1210653 PMC3016606

[36] Zhou H, Ivanov VN, Lien YC, Davidson M, Hei TK (2008) Mitochondrial function and nuclear factor-kappaB-mediated signaling in radiation-induced bystander effects. Cancer Res 68: 2233–2240. 18381429 10.1158/0008-5472.CAN-07-5278 PMC3715144

[37] Burdak-Rothkamm S, Short SC, Folkard M, Rothkamm K, Prise KM (2007) ATR-dependent radiation-induced gamma H2AX foci in bystander primary human astrocytes and glioma cells. Oncogene 26: 993–1002. 16909103 10.1038/sj.onc.1209863

[38] Burdak-Rothkamm S, Rothkamm K, Prise KM (2008) ATM acts downstream of ATR in the DNA damage response signaling of bystander cells. Cancer Res 68: 7059–7065. 18757420 10.1158/0008-5472.CAN-08-0545 PMC2528059

