# Supplementary figures and images for: The Biological Effect of Large Single Doses: A Possible Role for Non-Targeted Effects in Cell Inactivation
Source: PLoS One. 2014 Jan 22;9(1):e84991. doi: 10.1371/journal.pone.0084991 (PMC3898915; doi:10.1371/journal.pone.0084991)

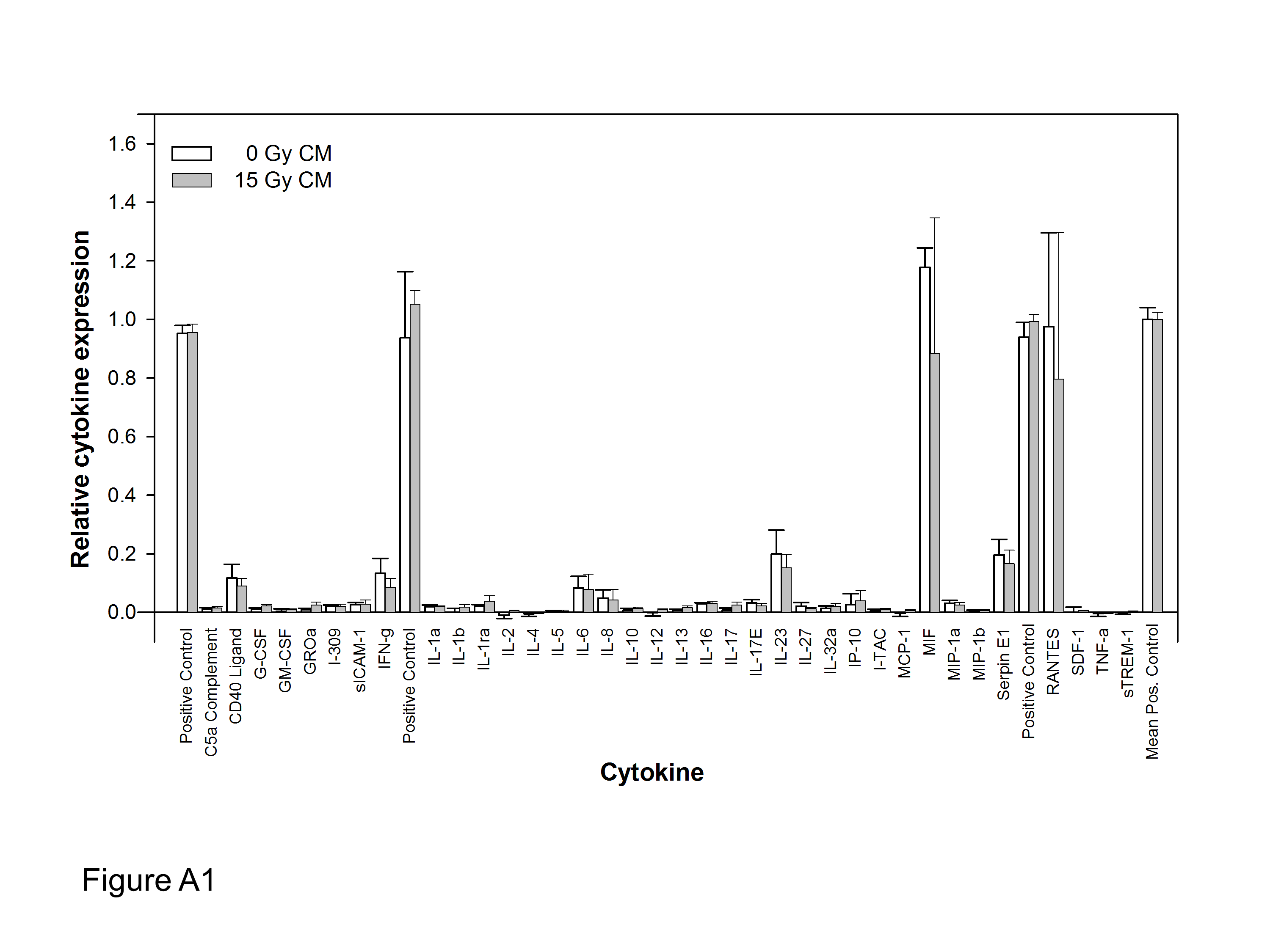

Supplement: Figure S1 — Relative protein levels in CM of all 36 cytokines on cytokine array. Mean values±standard errors of normalized signals of cytokines in CM from 15 Gy irradiated and unirradiated MCF7 cultures from three independent experiments are shown. No significant differences were observed between CM from irradiated and unirradiated cultures (P = 0.18–0.96; n = 3). MIF and RANTES showed high expression levels with considerable variation in between experiments (outliers). The variation did not appear to be limited to CM from irradiated cells (see data for RANTES). (TIF) [file pone.0084991.s001.tif]
